# *REM* Gene Family in Sugar Beet and Functional *BvREM12* in Salt Stress Tolerance

**DOI:** 10.3390/plants15182745

**Published:** 2026-09-08

**Authors:** Yan Liu, Jiajia Zhang, Xin Liu, Xiaoyu Sun, Huizi Duanmu

**Affiliations:** 1 Engineering Research Center of Agricultural Microbiology Technology, Ministry of Education, Heilongjiang University, Harbin 150080, China; liuyan200261@163.com (Y.L.); zhangjiajia1664@163.com (J.Z.); liuxin8924@163.com (X.L.); 2 Heilongjiang Provincial Key Laboratory of Ecological Restoration and Resource Utilization for Cold Region, School of Life Sciences, Heilongjiang University, Harbin 150080, China; 3 School of Information Technology, Zhongshan Polytechnic, Zhongshan 528400, China; sunxiaoyu@zspt.edu.cn

**Keywords:** salt stress, sugar beet, *BvREM12*, Remorin gene family, SOS signaling pathway

## Abstract

Soil salinization acts as a major abiotic stress and severely hampers global agricultural productivity. Elucidating the molecular mechanisms underlying plant salt tolerance is of great significance for crop improvement. Remorin (REM) proteins are plant-specific membrane marker proteins that play a key role in stress signaling; however, their function in the salt tolerance response of sugar beet remains unclear. In this study, 14 *BvREM* gene family members were identified. Phylogenetic analysis revealed that this family can be divided into five subgroups and exhibits significant interspecies collinearity with *Arabidopsis*. Transcriptomic analysis showed that several *BvREM* genes were up-regulated under salt stress, with *BvREM12* displaying greater expression fold change than most other family members. Using sugar beet line M14 as the gene resource material, we explored the potential function and proposed a working regulatory model for candidate gene *BvREM12* in salt stress response. Subcellular localization results indicated that the BvREM12 protein is localized to the cell membrane and nucleus. Overexpression of *BvREM12* significantly increased biomass accumulation in *Arabidopsis*, elevated antioxidant enzyme activities, decreased MDA levels, and increased proline content; a high K^+^/Na^+^ ratio was maintained, whilst Ca^2+^ levels were significantly higher than in the *Col-0*. Furthermore, qRT-PCR analysis revealed that several key salt-responsive genes were significantly up-regulated under salt stress. In summary, *BvREM12* integrates the ‘Ca^2+^-SOS3-SOS2-SOS1’ signaling pathway with the ‘H^+^-ATPase-AKT1’ ion transport system to synergistically regulate Na^+^ efflux and K^+^ influx, thereby maintaining cellular ion homeostasis and redox balance, and significantly enhancing the plant salt tolerance. This study systematically characterized the sugar beet *BvREM* gene family and functionally dissected *BvREM12*. Different from previous studies on REM proteins mainly focusing on ROS scavenging at plasma membrane nanodomains, our work reveals that *BvREM12*, which is dual-localized to the plasma membrane and nucleus, coordinates the Ca^2+^-dependent SOS signaling cascade and H^+^-ATPase-AKT1 ion transport system to jointly maintain ion homeostasis and redox balance under salt stress. This work provides candidate genes and a mechanistic reference for molecular breeding of salt-tolerant sugar beet.

## 1. Introduction

Soil salinization is one of the major environmental factors limiting global agricultural production; currently, approximately 7% of the world’s land area and 20–30% of irrigated farmland are threatened by salinization [1]. Salt stress inhibits plant growth and development and reduces crop yield through three mechanisms: osmotic stress, ionic toxicity and oxidative stress [2]. High concentrations of Na^+^ reduce soil water potential and induce stomatal closure, thereby limiting CO_2_ assimilation [3]; excess Na^+^ disrupts intracellular Na^+^/K^+^ homeostasis [4]; and it simultaneously induces a burst of reactive oxygen species (ROS), triggering secondary damage such as membrane lipid peroxidation [5]. At the molecular level, plants regulate ion homeostasis primarily via the Salt Over Sensitivity (SOS) pathway: salt stress elevates cytoplasmic Ca^2+^ levels; upon signal detection, SOS3 activates SOS2, which in turn phosphorylates the plasma membrane Na^+^/H^+^ antiporter SOS1 to drive Na^+^ efflux [6,7]. This process relies on the proton gradient established by the plasma membrane H^+^-ATPase (AHA1/2) to provide the driving force [8], and works in concert with the K^+^ influx channel AKT1 to maintain the K^+^/Na^+^ ratio [9]. Furthermore, plants eliminate excess ROS by activating antioxidant enzyme systems such as superoxide dismutase (SOD), peroxidase (POD), catalase (CAT) and ascorbate peroxidase (APX) [10]. They accumulate osmoregulatory substances such as proline to maintain turgor pressure [11]. Currently, elucidating the mechanisms of co-regulation between the SOS pathway and the antioxidant system has become a key area of research into plant salt tolerance. Remorins (REMs) are a key protein involved in this co-regulatory mechanism.

REM proteins are a family of plant-specific proteins whose homologues have been identified in all major land plant lineages, including ferns and mosses [12,13,14]. They are primarily associated with sterol- and sphingolipid-rich membrane rafts and are known plant pro-raft proteins [15]. REMs encode a family of oligomeric, filamentous proteins formed by coiled-coil structures, which are widely expressed in plant tissues such as roots, stems and leaves [16]. Their characteristic structural feature is a modular organization comprising a ‘conserved C-terminus + variable N-terminus’: the C-terminal domain contains a highly conserved coiled-coil structure and the hydrophobic membrane-anchoring motif REM-CA (REM C-terminal Anchor), which is the core region mediating protein oligomerization and plasma membrane anchoring [17,18,19], whilst the N-terminal domain is highly variable, rich in proline and containing potential isoprenylation sites, which participate in the regulation of protein conformational diversity [20]. Furthermore, the intrinsically disordered regions of REM proteins exhibit high rates of insertion/deletion mutations and phosphorylation modifications compared to their ordered regions [21]. These structural characteristics enable them to play a significant role in the perception and transduction of abiotic stress signals.

In recent years, genome-wide identification and functional studies of the *REM* gene family have been conducted in a variety of species, including wheat (*Triticum aestivum* L.) [22], tomato (*Solanum lycopersicum* L.) [13], foxtail millet (*Setaria italica* Beauv.) [23], poplar (*Populus trichocarpa*) [24], oilseed rape (*Brassica napus* L.) [25] and the endangered fern (*Adiantum nelumboides*) [26], among others. These studies indicate that REM proteins play a significant role in the plant response to abiotic stresses.

REM proteins play a central role in the plant response to salt stress by integrating hormonal signaling, regulating ion and ROS homeostasis, and maintaining cell membrane integrity. At the transcriptional level, the foxtail millet *SiREM6* is significantly induced by high salt stress. Its promoter contains DRE and ABRE elements and is regulated by a transcription factor SiARDP. Overexpression of this gene significantly enhances the germination rate, survival rate and proline content in transgenic *Arabidopsis* [23]; members of the tomato *SlREM* gene family respond to salt stress and treatment with various hormones, including ABA, MeJA and SA [13], whilst the oilseed rape *BnaREM1.3-4C-1* gene is able to complement the salt-sensitive phenotype of the *Arabidopsis rem1.3* mutant, indicating that *BnaREM1.3-4C-1* genes positively regulate salt tolerance via an ABA-dependent pathway [25]. At the physiological level, *Populus euphratica PeREM6.5* enhances the plant’s salt tolerance by up-regulating genes associated with water transport and ROS scavenging [27], whilst *Populus pubescens PtREM4.1/4.2/6.11* is up-regulated under salt stress and its promoter contains multiple stress response elements [24]. Together, these findings suggest that REM proteins confer synergistic salt tolerance by optimizing water transport and ROS scavenging. At the level of signal transduction and membrane stability, tomato *SlREM1* activates ROS bursts to trigger cell death, thereby participating in stress signal transduction [28], whilst the fern *AnREM* responds to abiotic stress by regulating ROS accumulation and stabilizing the cell membrane [26]. Furthermore, the differential expression of wheat *TaREM* during cold acclimation provides further support for the notion that REM proteins regulate ROS signaling through the organization of membrane microdomains, representing a conserved strategy across species [22]. In summary, REM proteins are induced by ABA and other hormones, with their expression regulated by transcription factors. On the one hand, they promote the accumulation of osmoregulatory substances and optimize water transport. On the other hand, they precisely regulate the balance between ROS burst and clearance whilst stabilizing cell membrane structure, ultimately enhancing plant salt tolerance in a systematic manner.

Sugar beet (*Beta vulgaris* L.) is the world’s second-largest sugar crop, accounting for ~20% of global sugar production [29]. It is also an important cash crop in China’s north-eastern region, with its roots possessing high economic value [30]. Although sugar beet possesses a certain degree of salt tolerance, the increasing prevalence of saline–alkali land worldwide has made crop security a major challenge [31]. The systematic identification of plant salt-tolerance genes to enhance crop stress resistance and increase yield is of great significance. Sugar beet M14 line [32,33] is a single chromosome-addition line obtained by backcrossing a diploid cultivated sugar beet (*Beta vulgaris* L.) with a tetraploid wild white-flowered sugar beet (*Beta corolliflora* L.). It possesses excellent traits such as apomixis and tolerance to salinity and drought. Although REM-mediated salt stress responses have been reported in multiple plant species, most previous studies primarily emphasized REM-dependent ROS scavenging and membrane-associated signal transduction. Few studies have explored whether REM proteins can integrate Ca^2+^-dependent SOS ion signaling pathways with H^+^-ATPase-driven ion transport modules, nor have they paid attention to the potential nuclear-associated functions of REM family members. Given that sugar beet M14 represents a special salt-tolerant monosomic addition line, we hypothesized that sugar beet-originated *BvREM12* may act as a multi-level signal integrator, coordinating both plasma membrane localized ion signaling events and potential nuclear-associated regulatory processes to confer salt tolerance. To test this hypothesis, we performed genome-wide identification of the *BvREM* gene family in sugar beet. We further characterized BvREM12 subcellular localization, overexpression phenotypes, physiological responses, as well as the expression dynamics of key ion homeostasis-related genes. Distinct from earlier REM studies in other plant species, our results highlight a potential synergistic regulatory model linking *BvREM12* to the Ca^2+^-SOS3-SOS2-SOS1 cascade and the H^+^-ATPase-AKT1 ion transport system. This work provides mechanistic insights into REM-associated salt tolerance responses in sugar beet germplasm, although further in planta validation in sugar beet is still required.

## 2. Results

### 2.1. Identification of the BvREM Gene Family

Using the hidden Markov model (HMM), 17 candidate genes were identified based on the C-terminal conserved domain, and three candidate genes were identified based on the N-terminal conserved domain. After removing redundant duplicate sequences, 14 *BvREM* entries were finally retained in the sugar beet genome. Among them, *BvREM3*/*BvREM4* and *BvREM6*/*BvREM7* are alternatively spliced isoforms derived from two genomic loci (LOC104888676 and LOC104889288). They were preserved as separate family members because their deduced protein sequences differ in amino-acid composition and length, which may produce functionally divergent protein isoforms, rather than encoding identical protein products. All 14 genes contain the REM_C conserved domain; of these, *BvREM4* and *BvREM8* also contain both the REM_C and REM_N conserved domains. It was found that the protein members encoded by the beet *REM* gene family exhibit conserved C-terminal structures, whilst their N-terminal structures are variable. The genes were arranged according to their chromosomal location and named *BvREM1–14*. Their gene names, sequence numbers, chromosomal localization, sequence start and stop positions, protein length, theoretical molecular weight, isoelectric point and predicted subcellular localization were compiled (Table S1).

Chromosomal distribution analysis showed that *BvREM* genes are unevenly distributed on six chromosomes (Chr1, Chr3, Chr5, Chr6, Chr7, and Chr8), with Chr1 and Chr6 containing the largest number of members, showing a clustered distribution pattern. The length of BvREM proteins ranges from 174 to 574 amino acids, with molecular weights from 19.32 kDa to 63.04 kDa and pI values ranging from 5.01 to 9.77. Most BvREM proteins are alkaline, while four members (BvREM5, BvREM6, BvREM7, BvREM8) are acidic or neutral proteins. Prediction of subcellular localization revealed that nine BvREM family members were localized to both the plasma membrane and nucleus, four were restricted to the plasma membrane, and only BvREM11 showed specific nuclear localization.

### 2.2. Phylogenetic and Collinearity Analysis of the BvREM Gene Family

**Figure 1:**
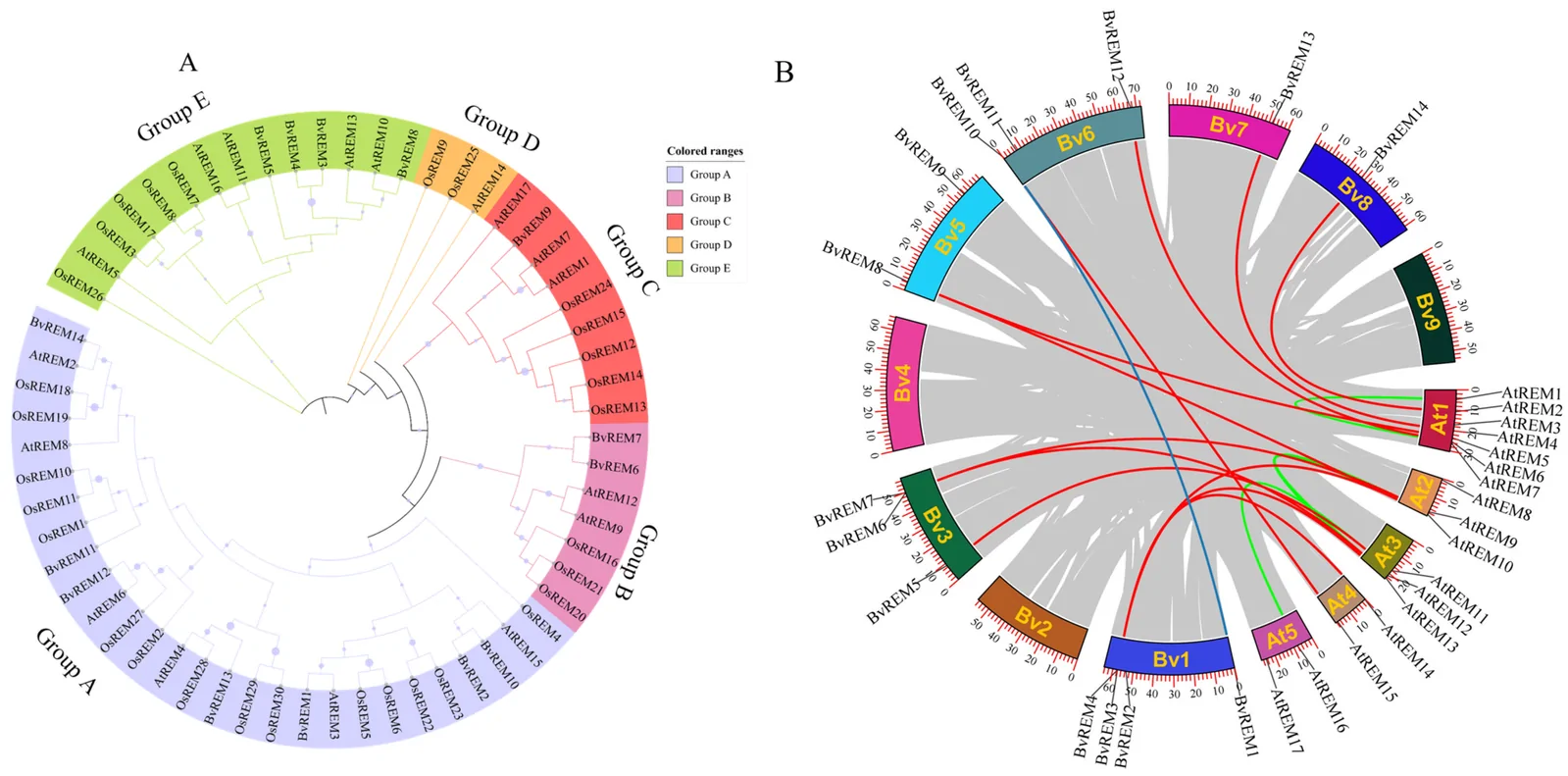
Phylogenetic and collinearity analyses of Remorin proteins. (**A**) A phylogenetic tree was constructed using the maximum likelihood method in MEGA software to elucidate the evolutionary relationships of Remorin family members in sugar beet (*Beta vulgaris* L.), *Arabidopsis thaliana*, and rice (*Oryza sativa*). In this phylogenetic tree, Remorin proteins were classified into five subgroups (A, B, C, D, and E), and each subgroup was distinguished by a different color. The parameters for sequence alignment were set as follows: for Pairwise Alignment, Gap Opening Penalty = 10 and Gap Extension Penalty = 0.1; for Multiple Alignment, Gap Opening Penalty = 10, Gap Extension Penalty = 0.2, and Gap Separation Distance = 0.4. All other parameters were set to the software defaults. The parameters for phylogenetic tree construction were set as follows: test of phylogeny: bootstrap method; number of bootstrap replications: 1500; model/method: Jones–Taylor–Thornton (JTT) model; rates among sites: uniform rates; gaps/missing data treatment: complete deletion; ML heuristic method: nearest-neighbor-interchange (NNI). (**B**) Collinearity analysis of *BvREM* genes. The central lines represent all segmentally duplicated genes in the genome, whilst the red, greenand blue lines connect *BvREM* genes that have undergone segmental duplication.

To investigate the evolutionary relationships and diversification patterns of the REM gene family across different plant species, a phylogenetic tree was constructed based on full-length REM protein sequences from sugar beet (*Beta vulgaris*, BvREM), *Arabidopsis thaliana* (AtREM), and rice (*Oryza sativa*, OsREM) (Figure 1A). Based on the topological structure of the maximum-likelihood tree, node bootstrap support values, and sequence homology among members, all REM protein members could be stably classified into five distinct monophyletic subgroups, designated Group A to Group E. Members within each subgroup clustered consistently with high branch support and a clear topological structure, indicating that the REM family has formed a relatively defined pattern of clade differentiation during evolution.

In terms of overall distribution, Group A is the largest core clade, containing the majority of REM members from the three aforementioned species, and is the most representative subgroup of this gene family. Group E is the second-largest clade, comprising 14 members from all three species (four sugar beet members, five Arabidopsis members, and five rice members), indicating that this branch is relatively conserved in both monocotyledons and dicotyledons. Group C contains nine members, most of which are from Arabidopsis and rice. Group B is a small clade with a total of seven members, evenly distributed across the three species (two members each in sugar beet and Arabidopsis, and three members in rice). Group D is the smallest clade, containing only three members (one Arabidopsis AtREM14 and two rice members, OsREM9 and OsREM25). No sugar beet homologous sequences were detected in this group, representing a small branch of species-specific differentiation. Regarding species distribution patterns, rice OsREM members are distributed across all five subgroups, showing the broadest evolutionary distribution. Arabidopsis AtREM members are also present in all five subgroups. In contrast, sugar beet BvREM members are only found in Groups A, B, C, and E, with no homologous sequences identified in Group D, reflecting the evolutionary divergence of this gene family among different species. Furthermore, REM proteins from different species within the same subgroup cluster stably. For example, both Group A and Group E contain multiple members from sugar beet, Arabidopsis, and rice, with clear paralogous and orthologous relationships, indicating that the *REM* gene family exhibits a high degree of evolutionary conservation across plant species.

There are multiple colinear segments among members of the beet *BvREM* family, with clear colinear relationships formed between some gene pairs, suggesting that they may have originated from genomic duplication events and have retained a high degree of sequence homology and conservation during evolution (Figure 1B). Furthermore, there is also a marked cross-species collinearity between the beet *BvREM* and *Arabidopsis AtREM* families, with multiple *BvREM* members forming colinear pairs with their *Arabidopsis* homologues. This indicates that the REM family is highly conserved during the evolution of dicotyledons, and that some of these colinear regions may harbor core functional genes.

In summary, phylogenetic clustering implies that such features may display putative evolutionary conservation among various plant species. Our findings offer valuable insights into the evolutionary origins, expansion patterns, and functional diversification of the *BvREM* gene family.

### 2.3. Analysis of Conserved Motifs, Promoters and Chromosomal Localization of the BvREM Genes

**Figure 2:**
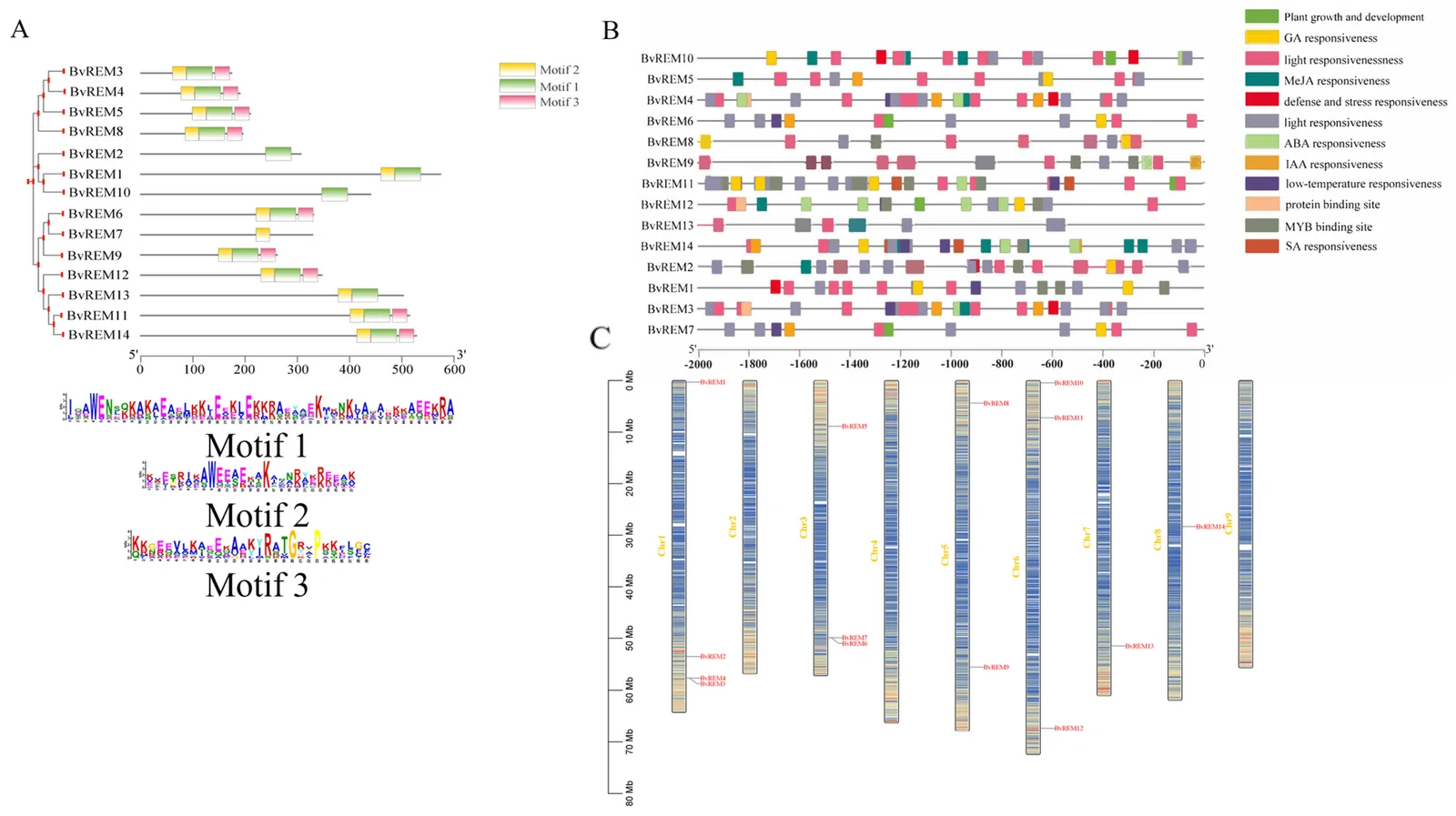
Analysis of conserved motifs, promoters and chromosomal localization of members of the *BvREM* gene family. (**A**) Distribution of conserved motifs in the *BvREM* gene family predicted using MEME; different motifs are marked with different colors. There are three highly conserved motifs, named Motif 1, Motif 2 and Motif 3, respectively. (**B**) Promoter regions covering the 2000 bp upstream of the start codon (ATG) of *BvREM* gene family members were extracted, and the PlantCARE database was used to predict promoter functional elements. (**C**) The chromosomal localization of *BvREM* genes is unevenly distributed, with members located on a total of six chromosomes: chromosomes 1, 3, 5, 6, 7 and 8.

The distribution characteristics of conserved motifs in the *BvREM* family revealed three highly conserved motifs identified via MEME analysis, named Motif 1, Motif 2 and Motif 3 respectively. Of these, Motif 1 (green) and Motif 2 (yellow) are present in the vast majority of BvREM proteins and represent the most central conserved motifs of the family (Figure 2A). Motif 3 (pink), however, is primarily found in BvREM3, BvREM4, BvREM5, BvREM6, BvREM8, BvREM9, BvREM11, BvREM12 and BvREM14, whilst BvREM1, BvREM2, BvREM7, BvREM10 and BvREM13 do not contain this motif, demonstrating distinct differences in motif composition.

From an overall distribution perspective, closely related *BvREM* members typically exhibit highly similar motif composition patterns. For example, BvREM3, BvREM4, BvREM5 and BvREM8 are situated on the same evolutionary branch; all contain the complete Motif 2–Motif 1–Motif 3 motif arrangement, and the positions and lengths of the motifs are also highly consistent; BvREM6 and BvREM9 also possess the Motif 2–Motif 1–Motif 3 combination, with only minor differences in sequence length. In contrast, members that are more distantly related exhibit marked diversification in motif composition; for instance, BvREM2 contains only Motif 1, BvREM1 contains only Motifs 2 and 1, BvREM10 contains only Motif 1, BvREM7 contains only Motif 2, and BvREM13 contains only Motif 2 and Motif 1. These differences reflect the divergent characteristics in the sequence structure of BvREM members across different lineages.

Analysis revealed that the *BvREM* gene promoter region contains a total of 13 types of functional elements (Figure 2B), which can be broadly categorized into three main groups: elements associated with plant growth and development, hormone response elements, and abiotic stress response elements. Among these, light-responsive elements are abundantly distributed across all members and constitute the most prominent feature of this family’s promoters; hormone-responsive elements include gibberellin (GA), methyl jasmonate (MeJA), auxin (IAA), salicylic acid (SA), abscisic acid (ABA) and defense and stress response elements. In addition, elements involved in stress adaptation and signal transduction, such as low-temperature response elements, MYB binding sites and protein binding sites, have also been identified.

In terms of distribution patterns, light-responsive elements are frequently found in the promoters of various *BvREM* members, suggesting that *BvREM* genes may be extensively involved in growth and development processes regulated by light signals. The distribution of hormone response elements, by contrast, exhibits marked diversity: MeJA response elements are abundant in most members, whilst GA, IAA and ABA response elements are selectively distributed across different members; SA response elements and some cold-responsive elements are present only in a few genes (such as *BvREM1*, *BvREM3* and *BvREM14*). It is worth noting that the promoter regions of several *BvREM* genes (such as *BvREM10*, *BvREM5* and *BvREM4*) contain a dense distribution of various types of response elements, suggesting that these genes may act as regulatory hubs, playing an important role in the complex crosstalk between environmental and hormonal signaling pathways.

Chromosomal localization analysis revealed that the *BvREM* genes are unevenly distributed across the beet genome, being localized on six chromosomes: 1, 3, 5, 6, 7 and 8; no *BvREM* members were detected on chromosomes 2, 4 or 9 (Figure 2C). Chromosome 1 harbors the largest number of members, including *BvREM1*, *BvREM2*, *BvREM3* and *BvREM4*; chromosome 3 contains *BvREM5*, *BvREM6* and *BvREM7*; chromosome 5 contains *BvREM8* and *BvREM9*; chromosome 6 contains *BvREM10*, *BvREM11* and *BvREM12*; chromosome 7 contains *BvREM13*; and chromosome 8 contains *BvREM14*. Some genes, such as *BvREM3* and *BvREM4*, and *BvREM6* and *BvREM7*, are located very close to one another on the chromosomes; these represent different alternatively spliced transcript isoforms from two single genomic loci, and they were annotated as separate family members due to differences in their predicted protein sequences.

In summary, the *BvREM* family exhibits a high degree of structural diversity and functional complexity in terms of motif composition, regulatory elements and chromosomal distribution. This provides an important molecular basis for its potential roles in light signal regulation, hormone response and stress adaptation, and lays the groundwork for subsequent functional characterization studies.

### 2.4. Analysis of the Expression Patterns of the BvREM Genes Under Salt Stress

**Figure 3:**
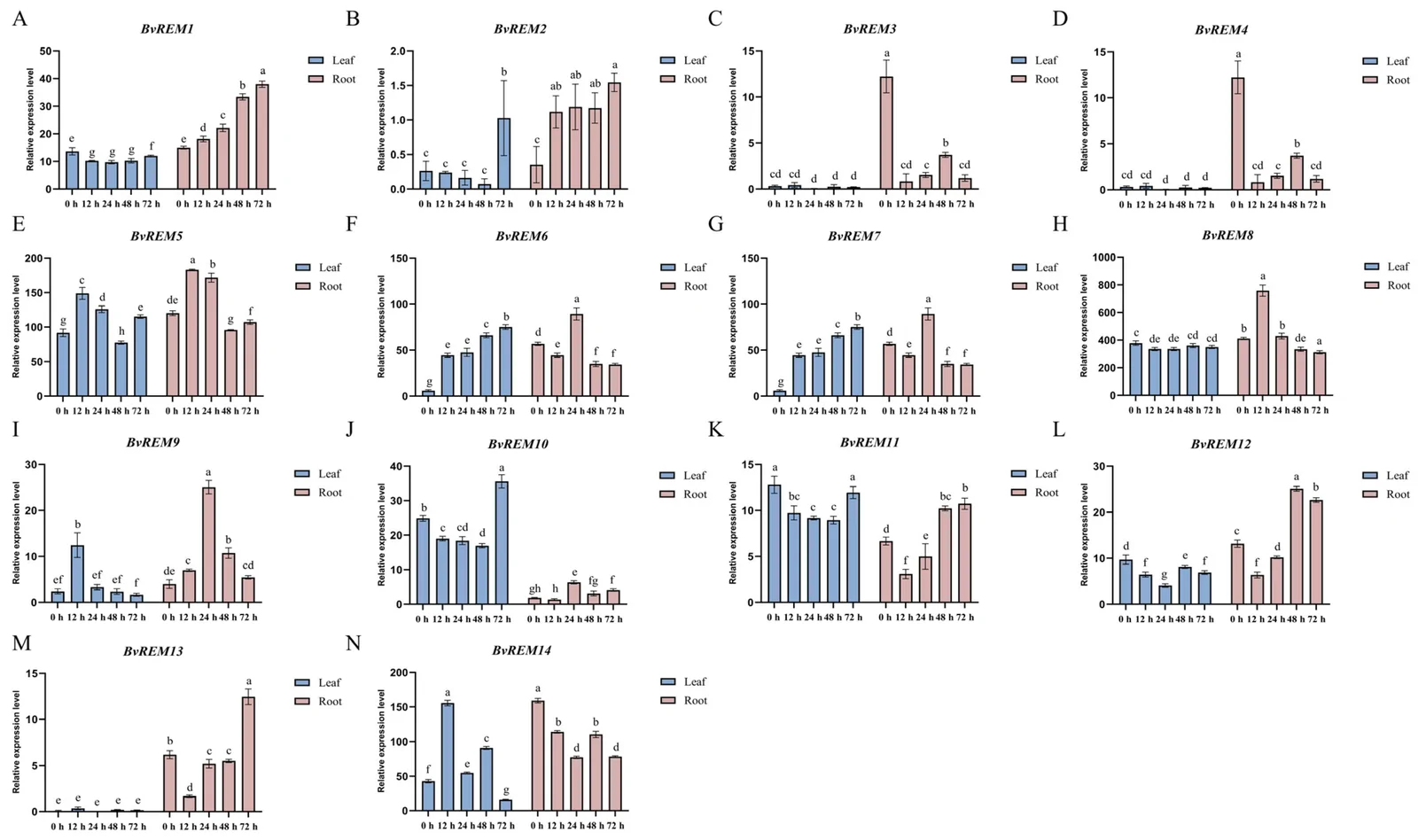
Expression profiles of 14 *BvREM* family genes under salt stress. Relative transcript levels of all *BvREM* members were detected in sugar-beet leaf and root tissues at 0 h, 12 h, 24 h, 48 h and 72 h after 150 mM NaCl treatment. Gene expression levels of (**A**) *BvREM1*; (**B**) *BvREM2*; (**C**) *BvREM3*; (**D**) *BvREM4*; (**E**) *BvREM5*; (**F**) *BvREM6*; (**G**) *BvREM7*; (**H**) *BvREM8*; (**I**) *BvREM9*; (**J**) *BvREM10*; (**K**) *BvREM11*; (**L**) *BvREM12*; (**M**) *BvREM13*; (**N**) *BvREM14*; in leaves and roots under salt treatment. Three independent biological replicates were included. Each bar represents mean ± standard error (SE). Columns labeled with different letters (a–h) indicate significant differences (*p* < 0.05). Notably, *BvREM3*/*BvREM4* and *BvREM6*/*BvREM7* are alternatively spliced isoforms derived from two genomic loci.

Based on previously published transcriptomic data of sugar beet under salt stress, we initially observed divergent expression patterns among members of the *BvREM* gene family upon salt treatment. To further verify their transcriptional changes, we analyzed the expression profiles of all 14 *BvREM* family members in root and leaf tissues of sugar beet at 0 h, 12 h, 24 h, 48 h and 72 h after 150 mM NaCl treatment (Figure 3). Distinct expression characteristics were exhibited by different family members; some *BvREM* genes were significantly up-regulated under salt stress, while the transcription of others was repressed or nearly unaffected by salinity.

Combining the above-mentioned bioinformatic results of the gene family and transcriptomic data, multiple lines of evidence support the selection of *BvREM12* as a high-priority candidate gene for subsequent functional studies: (1) phylogenetic analysis showed that *BvREM12* clustered within the same evolutionary clade as previously reported stress-responsive Remorin homologues from other plant species; (2) conserved-motif analysis revealed that *BvREM12* harbors the complete set of characteristic conserved motifs of the REM family; (3) promoter cis-element prediction identified numerous stress- and hormone-responsive cis-acting elements in its promoter region, implying that *BvREM12* may be subject to stress-related transcriptional regulation; (4) experimental results demonstrated that *BvREM12* was markedly induced by salt stress. Its transcript abundance in roots peaked at 24 h after salt treatment, reaching 1.9-fold relative to the 0 h control. Under identical salt stress conditions, its fold change in expression was higher than that of most other *BvREM* family members. This gene exhibited strong salt-inducible characteristics in both key tissues, and was therefore regarded as a candidate gene with great research potential. Collectively, these clues suggest that *BvREM12* is highly likely to participate in the salt stress response of sugar beet, motivating us to perform further functional experiments on this gene.

### 2.5. Subcellular Localization of BvREM12

**Figure 4:**
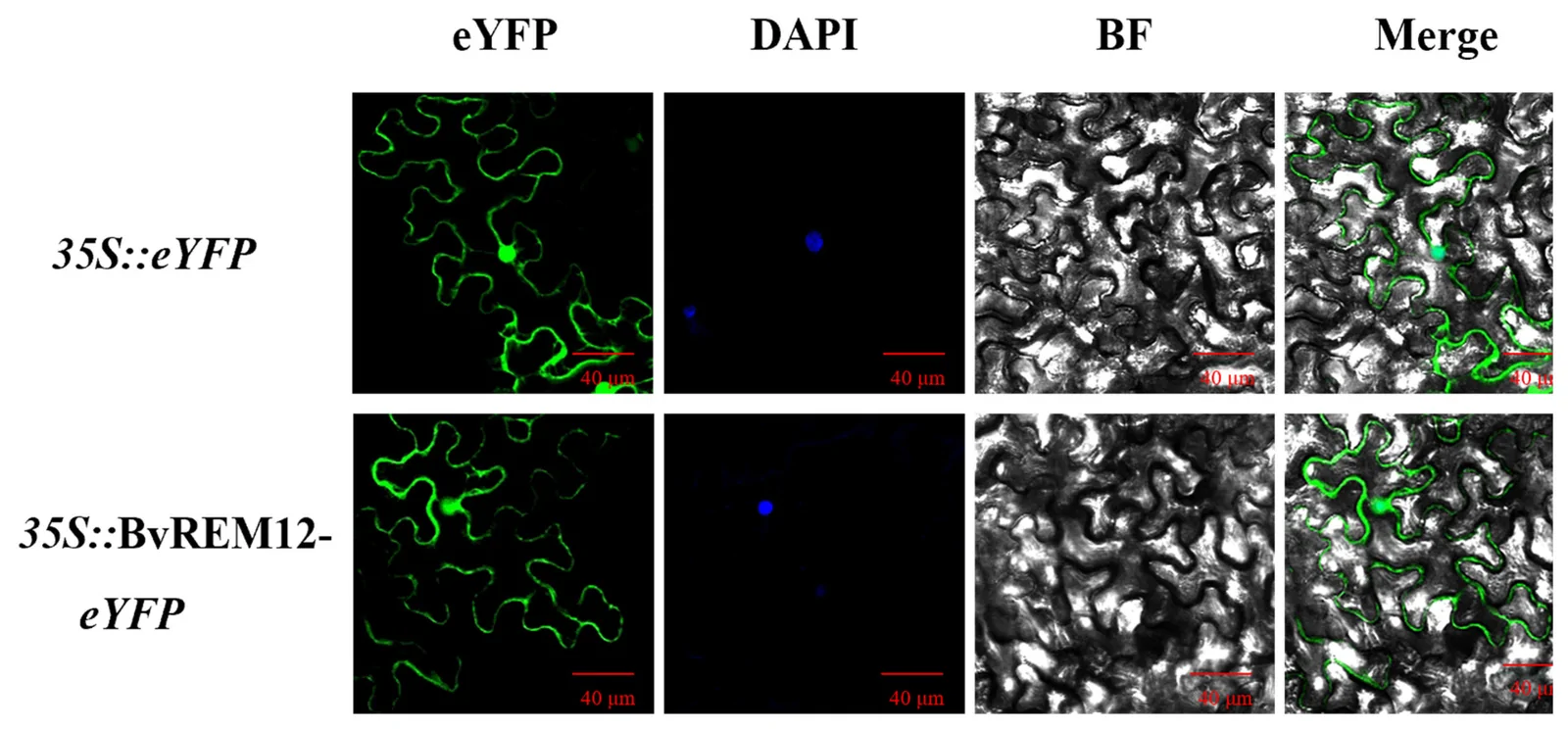
Subcellular localization of the BvREM12 protein in tobacco leaf epidermal cells. Subcellular localization of the BvREM12 protein and an empty vector in tobacco leaves. The free YFP signal expressed by the YFP empty-vector control was distributed in the cytoplasm and nucleus and overlapped with the nuclear localization dye signal. The BvREM12-eYFP fusion protein exhibited enriched fluorescence signals at the cell periphery (plasma membrane) besides the nuclear signal that colocalized with DAPI. The figure shows eYFP fluorescence, DAPI fluorescence (nuclear localization dye), bright-field image, and the merged image of bright field, eYFP fluorescence and DAPI fluorescence, respectively. Scale bar = 40 μm.

The subcellular localization of the BvREM12 protein was analyzed using eYFP imaging in Nicotiana benthamiana together with the nuclear staining dye DAPI (Figure 4). Confocal microscopy of the empty vector control revealed that free eYFP fluorescence was detected in both the cytoplasm and nucleus. Under identical experimental conditions, the green fluorescence of BvREM12-eYFP was observed in the nucleus, where it colocalized with DAPI signal, and was also present at the cell periphery (plasma membrane). We further examined the subcellular localization of BvREM12 in sugar beet protoplasts (Figure S2). Consistently, fluorescent signals were observed at the cell periphery. Subcellular localization prediction (Table S1) also supported that BvREM12 localizes to the plasma membrane and nucleus. Previous studies have reported that Remorin family proteins from *Populus euphratica* are specifically targeted to the plasma membrane [27], which represents a typical feature of Remorin proteins. Distinct from *Populus euphratica* REM proteins, BvREM12 exhibited dual localization to both the plasma membrane and the nucleus. This finding implies that, besides its potential role in sensing stress signals at the plasma membrane, BvREM12 may also participate in signal-regulatory processes within the nucleus.

### 2.6. Overexpression of BvREM12 Enhances Salt Tolerance in Arabidopsis

**Figure 5:**
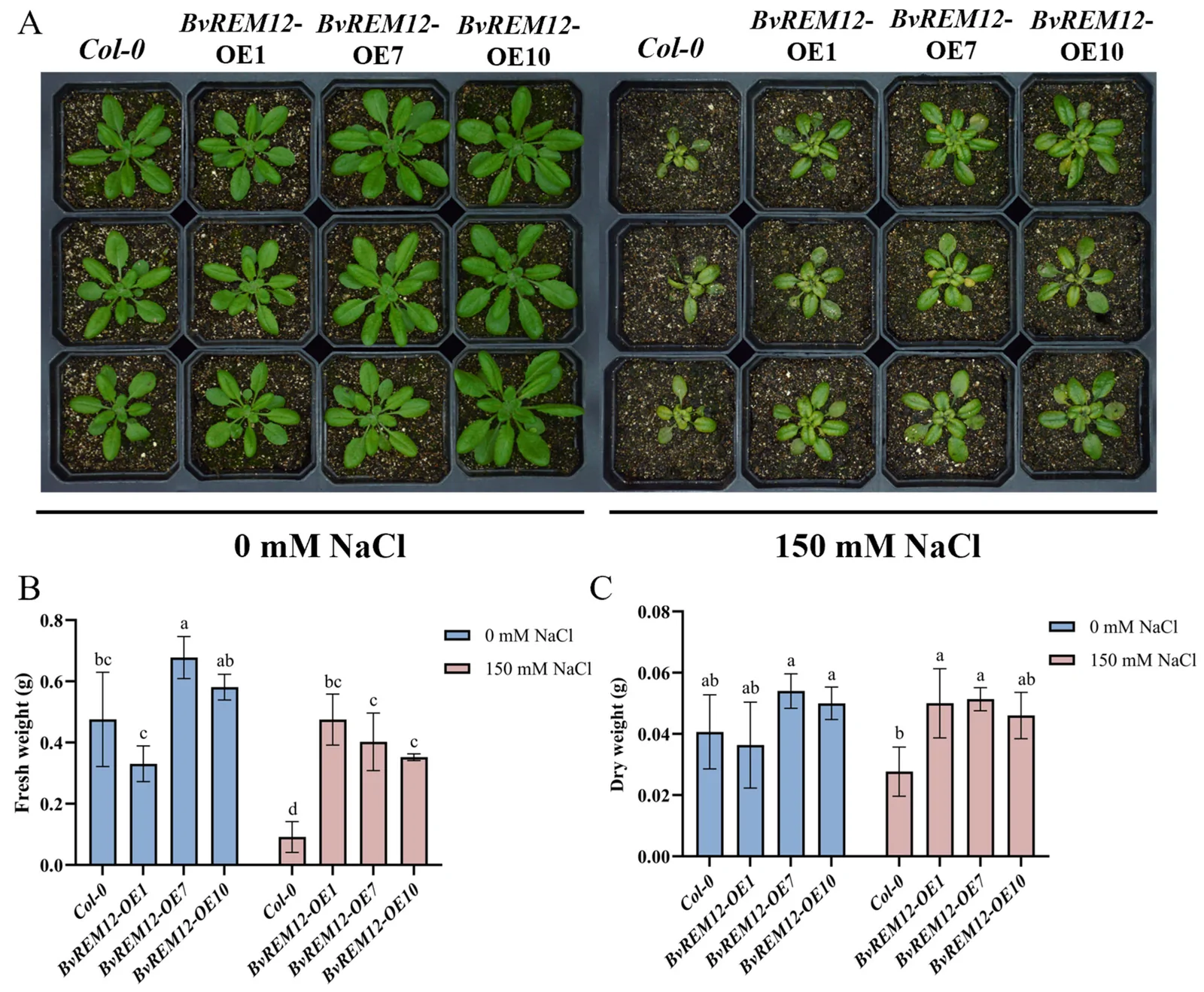
Phenotypic observations and dry and fresh weight results for *Col-0* and *BvREM12* overexpression (OE) plants under 0 and 150 mM NaCl treatments. (**A**) Under 0 mM NaCl, there was no significant difference in growth between *Col-0* and heterologously expressed plants; however, under 150 mM NaCl treatment, plant growth was inhibited and leaves exhibited symptoms such as wilting and yellowing. Nevertheless, the damage observed in the OE plants was less severe than that in the *Col-0* plants, indicating that the OE plants possess greater salt tolerance. (**B**) Under 0 mM NaCl treatment, the fresh weight of OE7 overexpressing plants was significantly higher than that of *Col-0* plants; under 150 mM NaCl treatment, the fresh weight of the heterologously expressed plants was significantly higher than that of the *Col-0* plants. (**C**) Under 0 mM NaCl conditions, no significant difference in dry weight was observed between overexpressing plants and *Col-0* plants; under 150 mM NaCl treatment, the dry weight of the heterologously expressed plants was significantly higher than that of the *Col-0* plants. Each column represents the mean of three independent replicates; error bars indicate standard error (SE). Columns labeled with different letters (a–d) indicate significant differences (*p* < 0.05).

*Col-0* and three independent *BvREM12* OE plants (OE1, OE7 and OE10) were examined. Under 0 mM NaCl, there was essentially no difference in the growth status of the *BvREM12* OE plants, which grew normally. Under 150 mM NaCl for 10 days, plant growth was significantly inhibited; the *Col-0* and three OE plants all exhibited wilting and yellowing. The *Col-0* plants showed more severe damage, with more pronounced leaf yellowing, greater curling, and more severe growth inhibition (Figure 5A).

Under 0 mM NaCl, the fresh weight of OE7 overexpressing plants was significantly higher than that of *Col-0* plants; under 150 mM NaCl, the fresh weight of *BvREM12* OE plants was significantly higher than that of the *Col-0*. Under 0 mM NaCl conditions, no significant difference in dry weight was observed between overexpressing plants and *Col-0* plants; under 150 mM NaCl conditions, the dry weight trend of *BvREM12* overexpressing plants relative to *Col-0* plants was consistent with the fresh weight trend (Figure 5B,C).

In summary, under salt stress conditions, the *BvREM12* gene promotes plant growth and increases plant biomass.

### 2.7. Physiological and Biochemical Analysis of the Overexpression Plants

Enzymatic activities of the plant antioxidant enzyme system and the levels of malondialdehyde (MDA) and proline (Pro) (associated with osmotic regulation) were measured.

**Figure 6:**
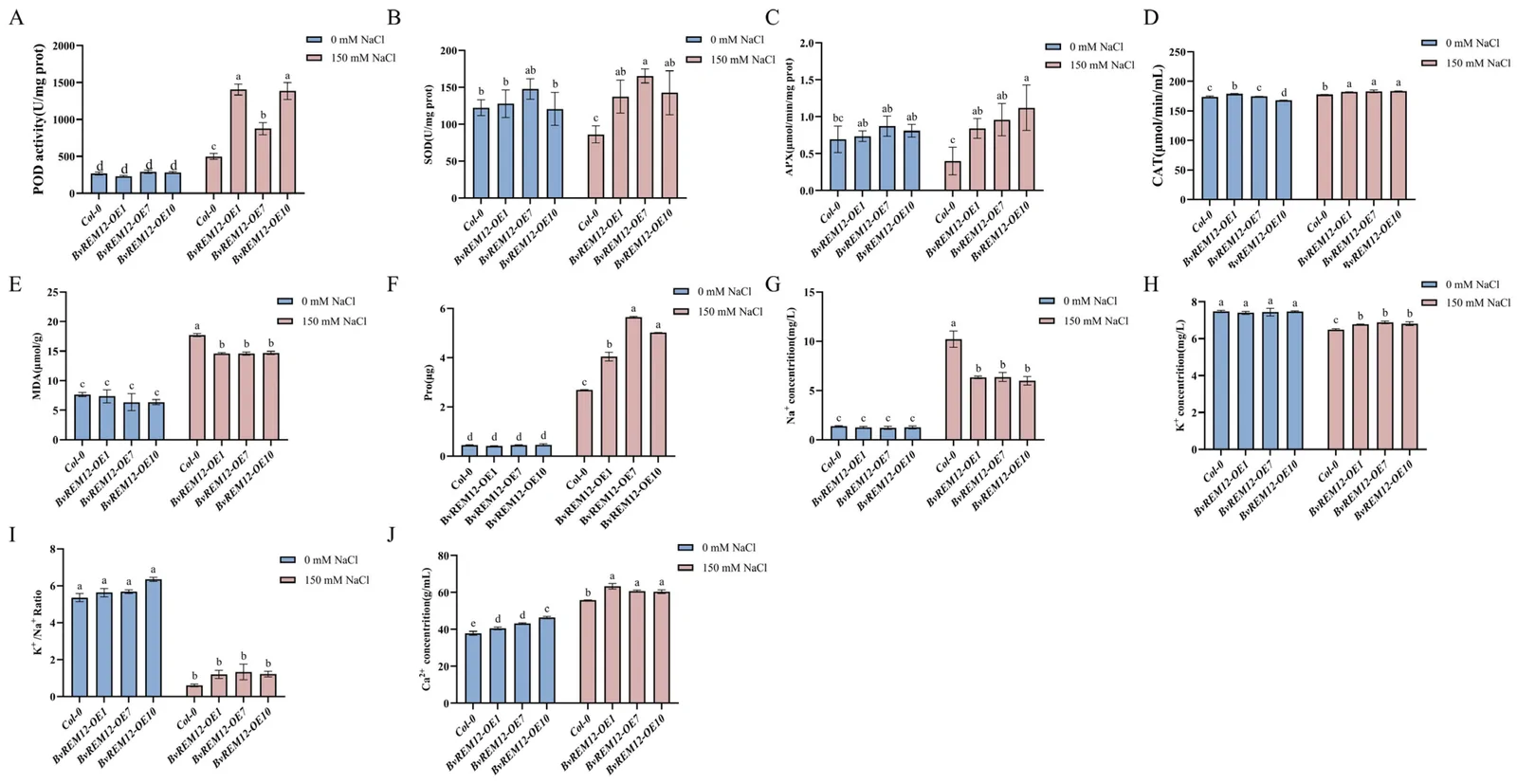
Physiological and biochemical parameters of *Col-0* and heterologously expressing plants under 0 and 150 mM NaCl treatment. (**A**) POD enzyme activity in *BvREM12* heterologously expressing plants and *Col-0* plants. (**B**) SOD activity in *BvREM12* OE plants and *Col-0* plants. (**C**) APX activity in *BvREM12* OE plants and *Col-0* plants. (**D**) CAT activity in *BvREM12* OE plants and *Col-0* plants. (**E**) MDA content in *BvREM12* OE plants and *Col-0* plants. (**F**) Proline content in *BvREM12* OE plants and *Col-0* plants. (**G**) Na^+^ content in *BvREM12* OE plants and *Col-0* plants. (**H**) K^+^ content in *BvREM12* OE plants and *Col-0* plants. (**I**) K^+^/Na^+^ ratio in *BvREM12* OE plants and *Col-0* plants. (**J**) Ca^2+^ content in *BvREM12* OE plants and *Col-0* plants. Each column represents the mean of three independent experiments; error bars indicate standard error (SE). Columns labeled with different letters (a–e) indicate significant differences (*p* < 0.05).

Under 0 mM NaCl, there were no significant differences between *BvREM12* OE plants and *Col-0* plants in terms of POD (Figure 6A), SOD (Figure 6B), APX (Figure 6C) and CAT (Figure 6D) enzyme activities, as well as MDA (Figure 6E) and Pro (Figure 6F) levels. Under 150 mM NaCl, the POD, SOD, APX and CAT enzyme activities, as well as Pro content, were significantly lower than those in the *BvREM12* OE plants, whilst MDA content was significantly higher than in *BvREM12* OE plants. The results indicate that under salt stress, the *BvREM12* gene positively regulates the activities of POD, SOD, APX and CAT in the antioxidant enzyme system, thereby maintaining intracellular ROS balance and minimizing oxidative damage; MDA is a key product of membrane lipid peroxidation induced by stress in plant cells and serves as an indicator of the extent of damage; higher MDA levels indicate greater damage to the plant. The results indicate that the *Col-0* plants suffered greater damage under salt stress; Pro, as one of the key osmoregulatory substances, plays a role in regulating cytoplasmic osmotic pressure. The results indicate that under salt stress conditions, the *BvREM12* gene can increase Pro accumulation in transgenic plants, thereby maintaining osmotic balance by regulating osmotic pressure and enhancing the salt tolerance of the transgenic plants.

Salt stress causes plants to suffer from ionic stress, leading to the accumulation of excessive Na^+^ within plant cells and disrupting the Na^+^ and K^+^ homeostasis; at the same time, Ca^2+^-mediated signal transduction triggers the plant’s response to salt stress (Figure 6G–J). In this experiment, the K^+^, Na^+^ and Ca^2+^ concentrations were measured in plants expressing the *BvREM12* gene heterologously and in *Col-0* plants. Under 0 mM NaCl treatment, there were no significant differences in Na^+^ and K^+^ concentrations or the K^+^/Na^+^ ratio between the OE plants and the *Col-0* plants; under 150 mM NaCl conditions, *BvREM12* OE plants exhibited significantly lower Na^+^ concentrations and significantly higher K^+^ concentrations compared to the *Col-0* plants, whilst maintaining a relatively high K^+^/Na^+^ ratio in both cases. Ca^2+^ levels in *BvREM12* OE plants were significantly higher than those in the *Col-0* both before and after salt treatment; however, following salt treatment, the increase in Ca^2+^ levels in the OE plants was more pronounced than in the *Col-0* plants. The results indicate that, under salt stress conditions, compared with *Col-0* plants, the *BvREM12* gene is able to reduce the damage caused by reactive oxygen species to plants, maintain cellular osmotic pressure, promote Na^+^ efflux, reduce K^+^ efflux, maintain a high K^+^/Na^+^ ratio within the plant, and enhance the plant’s salt tolerance. Furthermore, following salt stress, the *BvREM12* gene promotes an increase in Ca^2+^ levels, enhances signal transduction, activates relevant pathways, and triggers the plant response to salt stress.

### 2.8. Analysis of the Expression of Key Salt-Responsive Genes in Overexpression Plants

**Figure 7:**
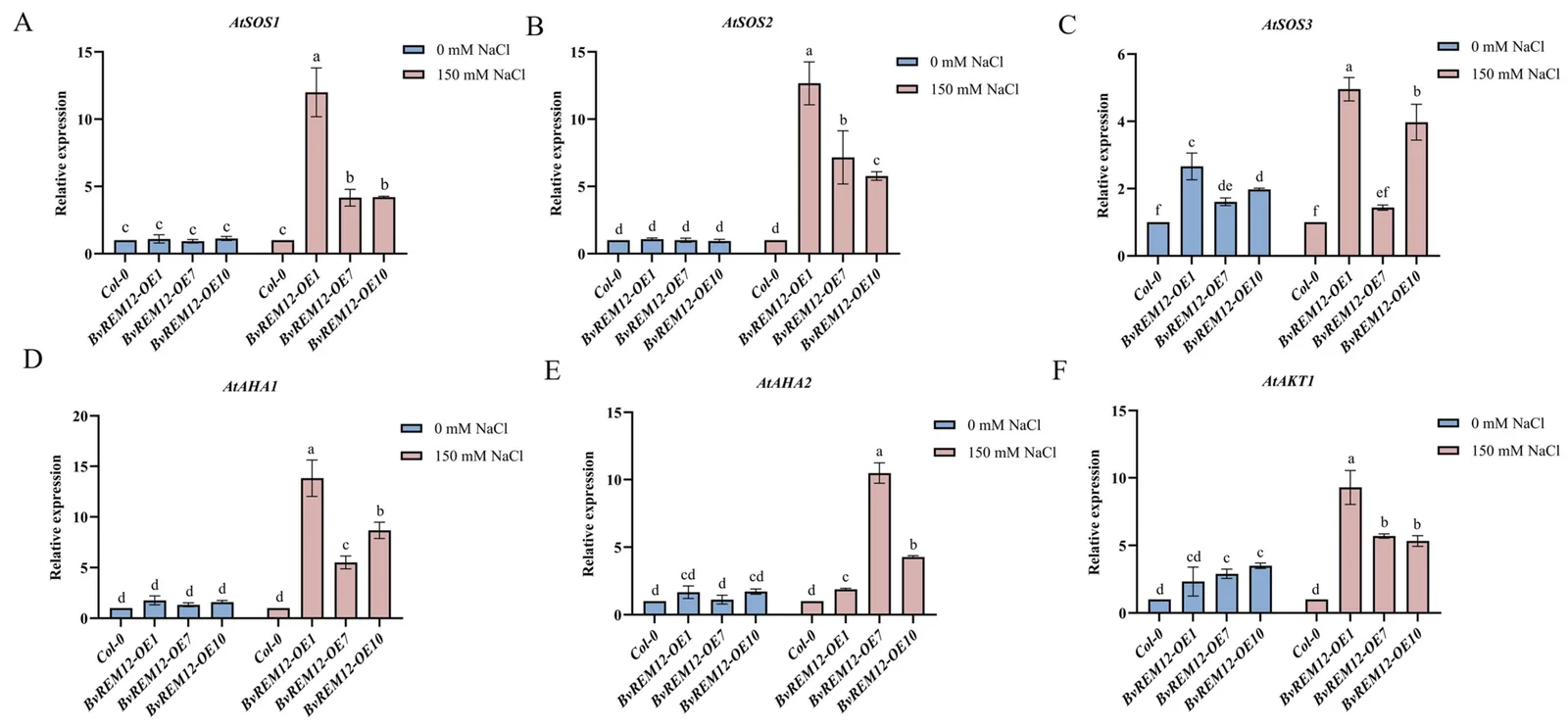
Analysis of salt stress-related gene expression in *BvREM12* OE plants. Analysis of relative expression levels of salt stress-related genes *AtSOS1* (**A**); *AtSOS2* (**B**); *AtSOS3* (**C**); *AtAHA1* (**D**); *AtAHA2* (**E**); *AtAKT1* (**F**) in *BvREM12* OE plants. Each column represents the mean of three independent experiments; error bars indicate standard error (SE). Columns labeled with different letters (a–f) indicate significant differences (*p* < 0.05).

Expression levels of key salt-responsive genes in the *BvREM12* OE plants and *Col-0* plants under 0 and 150 mM NaCl treatment are shown in Figure 7. Under 0 mM NaCl treatment, no significant differences were detected in the transcript levels of *SOS1* (Salt-Overly-Sensitive 1), encoding a Na^+^/H^+^ antiporter, and *SOS2* (Salt-Overly-Sensitive 2), encoding a Ser/Thr-protein kinase, between overexpressing (OE) and *Col-0* plants. However, the expression of the Ca^2+^-binding protein gene *SOS3* (Salt-Overly-Sensitive 3) was significantly higher than in the WT. Under 150 mM NaCl, the expression levels of the *AtSOS1*, *AtSOS2*, and *AtSOS3* genes in the OE plants were significantly higher than those in *Col-0* plants.

Under 0 mM NaCl, there was essentially no significant difference in the expression levels of the genes *AtAHA1* (Arabidopsis H^+^-ATPase 1) and *AtAHA2* (*Arabidopsis* H^+^-ATPase 2), which encode H^+^-ATPases, between the OE and *Col-0* plants. Under 150 mM NaCl, the expression levels of *AtAHA1* and *AtAHA2* in the OE plants were significantly higher than those in *Col-0* plants. Under 0 mM NaCl, the expression level of the *AtAKT1* (*Arabidopsis* K^+^-Transporter 1) gene, which encodes a K^+^ influx channel, was significantly higher in the OE plants than in *Col-0* plants; under 150 mM NaCl, the *AtAKT1* expression level in the OE plants was significantly higher than in *Col-0* plants.

The results indicate that, following salt stress, the *BvREM12* gene up-regulates the expression of *AtSOS1*, *AtSOS2*, *AtSOS3*, *AtAHA1*, *AtAHA2* and *AtAKT1*, thereby enhancing H^+^-ATPase activity. Through the SOS signaling pathway, it activates Na^+^/H^+^ antiporter and K^+^ influx channels, thereby participating in the regulatory process.

## 3. Discussion

This study identified 14 *BvREM* members in the beet genome with a conserved REM_C domain at the C-terminus and variable N-terminal domains. This finding is highly consistent with the classic structure of the REM protein family as plant-specific plasma membrane markers [14,15]. Phylogenetic results suggest that the basic subgroup structure of this family had already formed prior to the divergence of monocots and dicots [14]. Segmental duplication events may have driven family expansion, which is consistent with findings in species such as poplar [24] and millet [23]. It is worth noting that both *Bv*REM4 and *Bv*REM8 contain the REM_N and REM_C double domains, suggesting that they may retain relatively ancient ancestral characteristics. It is interesting to observe that the *BvREM* gene family is generally rich in elements associated with light response, hormone response (ABA, MeJA, SA, GA) and abiotic stress, suggesting that it may act as a signal integration hub, coordinating beet growth and development with responses to multiple abiotic stresses [22]. The subcellular localization of *BvREMs* to the plasma membrane and/or nucleus suggests that this family may play a role in both membrane signal transduction and nuclear regulation. This is consistent with recent research trends indicating that REM proteins are involved in transcriptional regulation within the nucleus [34].

Functional investigations of Remorin homologues across multiple plant species have demonstrated their important roles in abiotic stress adaptation. Heterologous overexpression of foxtail millet *SiREM6* in *Arabidopsis* enhanced salt tolerance by elevating proline accumulation and the survival rate under high-salinity conditions [23]. Ectopic expression of poplar *PeREM6.5* improved salt resistance by activating plasma membrane H^+^-ATPase activity and mitigating Na^+^-induced ionic injury [27]. In rapeseed, *BnaREM1.3-4C-1* could rescue the salt-sensitive phenotype of the *Arabidopsis rem1.3* mutant through an ABA-dependent regulatory pathway [25]. Transcriptomic evidence from tomato and poplar further demonstrated that many REM family genes are strongly responsive to salt, ABA and MeJA treatments, suggesting widespread involvement of REMs in hormone stress crosstalk [13,24]. Nevertheless, most of these earlier Remorin studies have largely concentrated on ROS scavenging, osmotic adjustment and membrane-associated stress signaling, while few reports have linked Remorin function to the Ca^2+^ dependent SOS ion signaling cascade together with H^+^-ATPase-mediated ion transport modules. Against this background, our study provides new inferential evidence connecting Remorin activity to these core salt-defense pathways.

Given the dual subcellular localization of BvREM12, we hypothesized that *BvREM12* might function as an organizational hub within plasma membrane nanodomains to assemble a putative “signalosome” containing ion transporters including SOS1, H^+^-ATPase and AKT1, thereby boosting salt stress signal transduction efficiency. Plasma membrane nanodomains are well known to serve as specialized micro-compartments for concentrating signal components to accelerate signal perception and transduction in plant cells [14,15]. Similar scaffold-like regulatory modes have been proposed for several membrane-associated plant proteins under abiotic stress. Nevertheless, it must be emphasized that this is only a hypothetical working model inferred from our physiological and qRT-PCR datasets, rather than a biochemically proven regulatory cascade. In addition, although multiple stress-related cis-acting elements were predicted within the *BvREM12* promoter by PlantCARE, these remain purely in silico predictions. Further promoter–reporter assays (e.g., promoter GUS or dual luciferase transient expression assays) will be required in future work to experimentally verify the salt-responsive promoter activity of *BvREM12*. Direct biochemical evidence from protein–protein interaction assays, such as co-immunoprecipitation (Co-IP), bimolecular fluorescence complementation (BiFC), or surface plasmon resonance (SPR), will be required in future work to verify whether *BvREM12* physically interacts with these ion transport proteins. In addition, although multiple pieces of evidence including tobacco transient expression, sugar beet protoplast assay and in silico prediction support dual plasma-membrane and nuclear localization of BvREM12, further co-localization experiments using bona fide plasma-membrane fluorescent markers combined with quantitative co-localization analysis are required in future work to provide more solid experimental evidence for its plasma-membrane targeting.

It is worth highlighting that all phenotypic, physiological and molecular functional characterization in this work relies on heterologous overexpression in *Arabidopsis*. Salt stress responses frequently exhibit pronounced species-specific differences [35,36]. Accordingly, results obtained from the heterologous *Arabidopsis* system cannot fully reflect the authentic endogenous function of *BvREM12* in salt-tolerant sugar beet M14. At present, stable genetic transformation and gene editing workflows for sugar beet M14 remain technically challenging in our laboratory. Therefore, further reverse genetic experiments are needed, such as gene knockout or virus-induced gene silencing (VIGS) in sugar beet, as well as complementation tests using *Arabidopsis* Remorin loss-of-function mutants, to validate the in vivo physiological role of endogenous *BvREM12*. These validations will be prioritized in our future research, and the regulatory cascade presented herein should be interpreted as a potential working model rather than a fully elucidated definitive mechanism.

Cumulative evidence from halophyte research indicates that SOS-dependent Ca^2+^ signaling, plasma membrane H^+^-ATPase activity and AKT1-mediated K^+^ uptake cooperatively sustain cellular ion homeostasis under salinity [37,38]. In halophytic crops, activation of the SOS cascade and proton pump systems is essential for extruding excess Na^+^ and preserving a favorable K^+^/Na^+^ ratio to alleviate ionic toxicity [24]. Combined with physiological and molecular data from heterologous overexpression materials, our results infer that *BvREM12* can positively modulate these core salt defense modules, together with antioxidant defense and osmotic adjustment systems. This is broadly consistent with earlier Remorin-mediated salt tolerance reports in woody and herbaceous plants [2,28]. Even so, it remains unclear how *BvREM12* modulates upstream Ca^2+^ signaling output to trigger SOS pathway activation, which merits further investigation.

Apart from its plasma-membrane localization, BvREM12 is also detected in the nucleus. The nuclear pool of BvREM12 raises an intriguing possibility that Remorin family proteins may participate in transcriptional reprogramming during abiotic stress responses. Multiple transcriptomic studies have predicted nuclear targeting for various REM isoforms; however, direct experimental evidence supporting nuclear REM-dependent stress regulation is still limited [39]. This opens a promising future research direction to dissect nucleocytoplasmic shuttling and nuclear-dependent regulatory functions of plant Remorins.

## 4. Materials and Methods

### 4.1. Plant Materials and Treatments

The *Arabidopsis thaliana* ecotype ‘Columbia-0’ (*Col-0*) and *BvREM12* OE seeds were sown one seed per pot into the mixed soil substrate (soil and vermiculite were mixed at 1:1) and cultured (22 °C, 16 h light/8 h dark). Following 21 days of cultivation, plants entered the vigorous growth phase. *Col-0* and *BvREM12* OE plants were treated with 150 mM NaCl via root irrigation for 10 days. The treatment group was supplied with 30 mL of NaCl solution daily, and the control group was irrigated with an equivalent volume of water. Leaves were collected from three uniformly sized individuals per line for physiological–biochemical measurements and quantitative real-time PCR (qRT-PCR) experiments.

*Nicotiana benthamiana* seedlings were cultivated in a growth chamber at 22 °C under a 16 h light/8 h dark photoperiod. Seedlings that had developed at least three pairs of true leaves were selected for Agrobacterium-mediated infiltration treatment.

### 4.2. Genome-Wide Identification of the BvREM Gene Family

The whole-genome protein sequences of sugar beet were retrieved from the NCBI database (https://www.ncbi.nlm.nih.gov) (accessed on 10 January 2026). The hidden Markov model (HMM) files of REM conserved domains (PF03766 for N-terminal domain and PF03763 for C-terminal domain) were downloaded from the InterPro database (https://www.ebi.ac.uk/interpro/) (accessed on 15 January 2026). The hmmsearch program of HMMER was used to screen candidate REM proteins from the sugar beet proteome. Candidate sequences were further verified using the SMART database (https://smart.embl.de) (accessed on 26 January 2026) to confirm the integrity of conserved domains, and redundant sequences without typical REM domains were eliminated. All identified *BvREM* genes were named according to their chromosomal locations.

### 4.3. Sequence and Gene Structure Analysis of BvREM Family Members

The ProtParam tool on the ExPASy platform (https://www.expasy.org/resources/protparam) (accessed on 3 February 2026) was used to predict the physicochemical properties of BvREM proteins, including molecular weight and theoretical isoelectric point (pI). TBtools-II (Version 2.0; South China Agricultural University, Guangzhou, China) was applied to extract chromosomal location, coding sequence length and amino acid number of each *BvREM* gene. The Plant-mPLoc (www.csbio.sjtu.edu.cn/bioinf/plant-multi/) (accessed on 3 February 2026) server was used for subcellular localization prediction of BvREM proteins.

### 4.4. Phylogenetic Analysis of the BvREM Gene Family

The genomic, proteomic, and genome annotation (GFF) files of sugar beet (*Beta vulgaris* L.) were retrieved from the National Center for Biotechnology Information (NCBI) database (https://ncbi.nlm.nih.gov) (accessed on 15 February 2026). The genome assembly versions used were sugar beet EL10.2 (GCA_026745355.1), *Arabidopsis thaliana* TAIR10.1 (GCA_000001735.2), and rice (*Asian cultivated rice*) AGIS1.0 (GCA_034140825.1). Hidden Markov model (HMM) profiles for the characteristic conserved domains of the *REM* gene family Remorin N-terminal region (Pfam accession: PF03766) and Remorin C-terminal region (Pfam accession: PF03763) were downloaded from the InterPro database (https://www.ebi.ac.uk/interpro/entry/pfamPF03766/) (accessed on 21 February 2026). Using HMMER 3.0 software, the HMM profiles of PF03766 and PF03763 were applied to perform homology searches against the local sugar beet proteome database with an E-value cutoff of <0.05 to obtain candidate REM protein sequences. All candidate sequences were subjected to multi-database conserved-domain annotation and rigorous validation using the Simple Modular Architecture Research Tool (SMART, https://smart.embl.de/) (accessed on 6 March 2026) and the NCBI Conserved Domain Database search tool CD-Search (https://www.ncbi.nlm.nih.gov/Structure/cdd/wrpsb.cgi) (accessed on 11 March 2026). After manual screening based on complete conserved-domain architectures, only candidate sequences with both Remorin N-terminal region and Remorin C-terminal region domains detected in all three aforementioned databases were retained and finally identified as bona fide members of the sugar beet *REM* gene family. Multiple sequence alignment of REM proteins from sugar beet, *Arabidopsis thaliana*, and rice was performed using MEGA 5.0.1.102 software, and a maximum-likelihood phylogenetic tree was subsequently constructed.

### 4.5. Analysis of Conserved Motifs and Domains of BvREM Family

Genomic sequences and CDS sequences of *BvREM* genes were submitted to the Gene Structure Display Server 2.0 to visualize exon–intron structures (https://gsds.gao-lab.org/) (accessed on 13 March 2026). Conserved domains were annotated via the SMART database. The MEME online tool (https://meme-suite.org/meme/db/motifs) (accessed on 22 March 2026)was used to identify conserved motifs of BvREM proteins. TBtools was used to integrate and visualize gene structures, conserved domains and conserved motifs.

### 4.6. Chromosomal Localization and Collinearity Analysis

Chromosomal information, whole genome sequences and GFF annotation files of sugar beet were obtained from NCBI. The MCScanX algorithm was adopted to analyze intra-genomic collinearity of sugar beet and inter-genomic collinearity between sugar beet and *Arabidopsis*. Chromosomal distribution and collinearity relationships of *BvREM* genes were visualized by TBtools.

### 4.7. Promoter Cis-Element Analysis

Sequence extraction was performed using the 2000 bp region upstream of the start codon of each *BvREM* gene’s CDS as the promoter region; the resulting sequences were submitted to the PlantCARE online database (https://bioinformatics.psb.ugent.be/webtools/plantcare/html/) (accessed on 27 March 2026), where the system analyzed the types, numbers and distribution characteristics of the cis-acting elements contained within the promoter region.

### 4.8. Analysis of the Expression Patterns of the BvREM Gene Family

RNA-seq data from salt-stressed beet leaves and roots were downloaded from the NCBI SRA database (accession number: PRJNA666117). This is a publicly available dataset derived from diploid sugar beet (*Beta vulgaris* L.) subjected to abiotic stress treatments. This dataset was exclusively employed for in silico screening of candidate *BvREM* genes, and all subsequent experimental validations were carried out using the sugar beet M14 line. Expression data for the *BvREM* genes family were screened, and bar charts were generated using GraphPad Prism (Version 10.1.2; GraphPad Software, LLC, San Diego, CA, USA).

### 4.9. Subcellular Localization Assay

To determine the subcellular localization of the BvREM12 protein, the beet M14 strain was used as the experimental material. Specific primers were designed to clone the full-length CDS sequence of *BvREM12*, and the CDS sequence was cloned into the eukaryotic expression vector pCAMBIA2300-*35S::*eYFP [40] via homologous recombination, yielding pCAMBIA2300-*35S::BvREM12*-eYFP. All primer sequences and instruments are listed in Supplementary Tables S2 and S4. The recombinant plasmid was transformed into Agrobacterium *GV3101*, and the bacterial suspension was injected into tobacco leaves using the Agrobacterium transient transformation method. The leaves were kept in the dark for 24 h, followed by 24 h of light exposure, after which samples were collected and the fluorescent signals in the tobacco leaves were observed under a confocal microscope.

### 4.10. Observation of Phenotypes in Heterologously Expressing Plants and Determination of Physiological and Biochemical Parameters

To construct *BvREM12* overexpression (OE) plants, the CDS sequence of *BvREM12* was cloned into the eukaryotic expression vector pCAMBIA1300-*35S::3 × FLAG* [40] via homologous recombination, yielding pCAMBIA1300-*35S::3 × FLAG-BvREM12*; this was transformed into Agrobacterium *GV3101* and used to infect *Arabidopsis* plants via the floral dip method [41]. Afterwards, T0 seeds from *Arabidopsis* heterologously overexpressing *BvREM12* were collected. The seeds were disinfected with three rinses of 75% ethanol followed by one rinse of absolute ethanol, and plated onto half-strength MS medium supplemented with 30 mg/L hygromycin. Seedlings that developed two pairs of true leaves were transplanted into soil. Leaf samples were harvested and assayed at DNA, RNA and protein levels to identify T3-generation positive transformants (Figure S1). Subsequent experiments were performed using T3-generation plants.

Assays for MDA content, SOD activity, POD activity, CAT activity and APX activity were conducted using kits purchased from Suzhou Grees Biotechnology Co., Ltd., Suzhou, China. The specific procedures were carried out in accordance with the instructions provided with the respective kits.

Determination of Na^+^, K^+^ and Ca^2+^ concentrations was done using flame atomic absorption spectrophotometry [42].

### 4.11. Quantitative Real-Time PCR (qRT-PCR)

Leaves were harvested from three uniformly grown individuals per line and stored at −80 °C for subsequent RNA isolation and qRT-PCR assays. Total RNA was extracted from salt-treated plant material using the TRIzol method [43]. Denatured RNA was reverse-transcribed according to the instructions of the Takara reverse transcription kit, with the reaction program set to 37 °C for 15 min and 80 °C for 5 s. Using the reverse-transcription products as templates, qRT-PCR amplification was performed with gene-specific primers to analyze the expression of *BvREM12* in *Arabidopsis*, with Actin (NM_001338359) serving as the internal control. The expression of selected salt-responsive genes in *BvREM12* OE plants was also analyzed using the same procedure.

The total qRT-PCR reaction volume was 20 μL, comprising 15 μL of SYBR, 2 μL of cDNA, 0.6 μL each of the forward and reverse primers, and RNase-free ddH_2_O added to a final volume of 20 μL. The qRT-PCR program was as follows: 95 °C for 3 min, 95 °C for 5 s, 57 °C for 35 s, repeated for 35–40 cycles, followed by 95 °C for 15 s, 60 °C for 1 min, 95 °C for 30 s, and 60 °C for 15 s. Each sample was analyzed in triplicate. Data were analyzed using the 2^−ΔΔCt^ method [44]. All primer sequences and instruments are listed in Supplementary Tables S2 and S4.

### 4.12. Data Analysis

All experimental data were obtained from at least three independent biological replicates and processed using GraphPad Prism (Version 10.1.2; GraphPad Software, LLC, San Diego, CA, USA) and SPSS Statistics (Version 31.0; IBM Corporation, Armonk, NY, USA). All results are presented as mean ± standard deviation (SD). Statistical differences among groups were evaluated using one-way analysis of variance (ANOVA) followed by Duncan’s multiple range test, with *p* < 0.05 set as the threshold for statistical significance.

## 5. Conclusions

**Figure 8:**
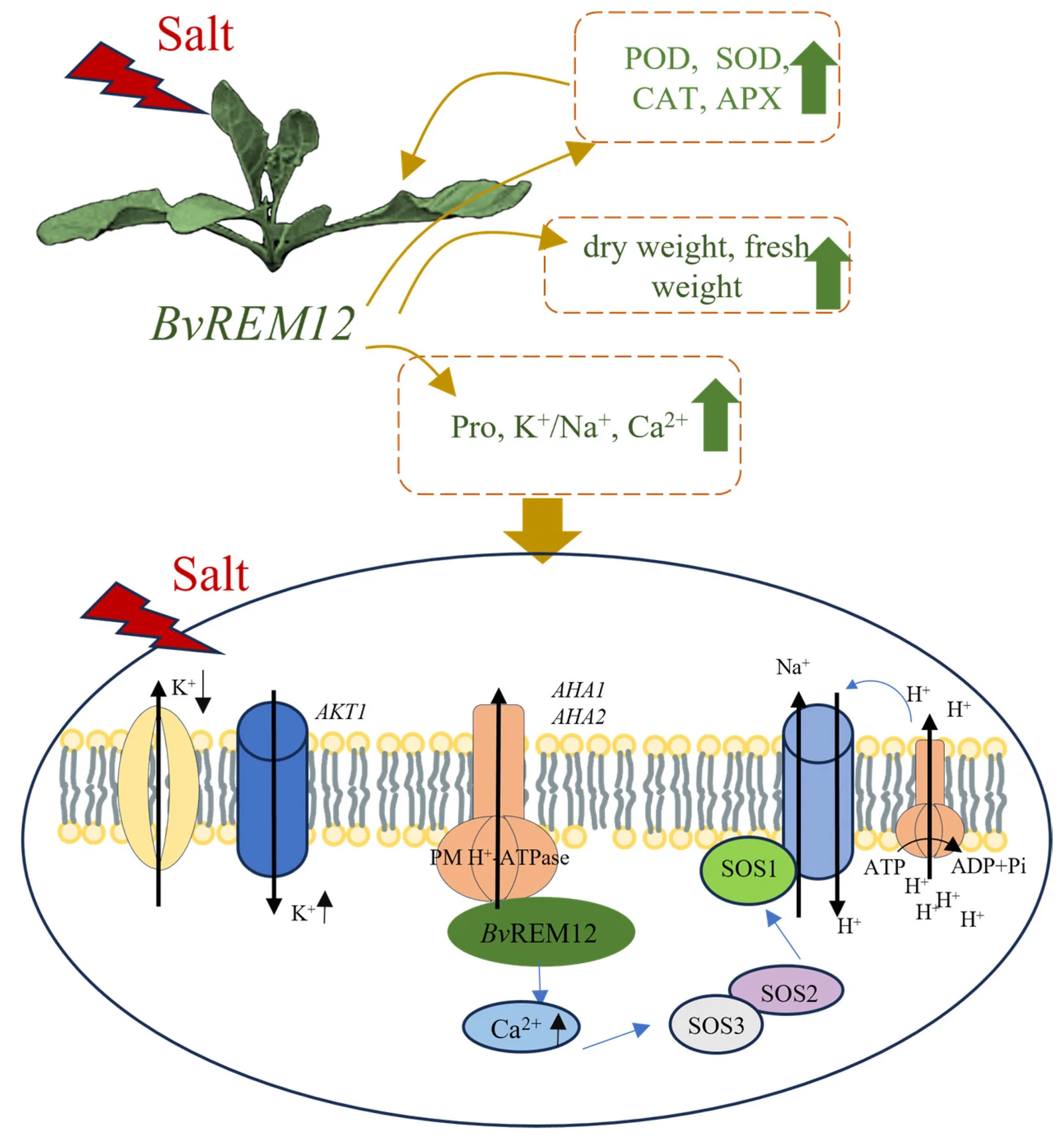
Schematic diagram illustrating a working model for *BvREM12*-mediated salt tolerance responses derived from heterologous overexpression data. *BvREM12* is a key regulator of plant salt tolerance that enhances salinity resistance through multiple coordinated mechanisms. Under salt stress conditions, *BvREM12* increases the activities of antioxidant enzymes including POD, SOD, CAT, and APX, thereby alleviating oxidative damage. Simultaneously, it promotes proline accumulation for osmotic adjustment while increasing the K^+^/Na^+^ ratio and Ca^2+^ content to maintain ion homeostasis. At the cellular level, *BvREM12* modulates plasma membrane H^+^-ATPase (PM H^+^-ATPase) activity and activates the SOS signaling pathway (SOS1-SOS2-SOS3) to drive Na^+^ efflux, facilitates K^+^ uptake via the AKT1 channel, and sustains proton gradients through AHA1/AHA2, collectively improving plant salt tolerance and biomass accumulation.

Genome-wide bioinformatics analysis of the *BvREM* gene family in sugar beet M14 has not been previously reported. It led to the functional characterization of the *BvREM12* gene in plant response to salt stress. The main conclusions are as follows:

(1) A total of 14 members of the *BvREM* gene family across the entire beet genome were revealed. (2) Heterologous overexpression of *BvREM12* improved salt stress tolerance in *Arabidopsis*. (3) This salt-tolerant phenotype was associated with enhanced antioxidant capacity, elevated osmotic-adjustment levels, and improved maintenance of ion homeostasis. Our results based on heterologous overexpression support a potential working model linking *BvREM12* to the SOS-related salt tolerance pathway (Figure 8). *BvREM* promotes Na^+^ efflux by enhancing Ca^2+^ signaling to activate the SOS3-SOS2-SOS1 cascade; simultaneously, by increasing H^+^-ATPase activity to provide the proton driving force for SOS1 and activating *AKT1*-mediated K^+^ influx, thereby synergistically maintaining cellular ion homeostasis. We also note that the signalosome-assembly hypothesis requires biochemical protein interaction assays for further experimental validation.

## Data Availability

The original contributions presented in this study are included in the article/Supplementary Materials. Further inquiries can be directed to the corresponding author.

## Supplementary Materials

The following supporting information can be downloaded at https://www.mdpi.com/article/10.3390/plants15182745/s1, Table S1: *BvREM* Gene Family Characteristics; Table S2: List of the primers used in the study; Table S3: *BvREM* Gene Transcriptome Data; Table S4: List of instruments for the experiment; Figure S1: Molecular identification of *BvREM12*-overexpressing T3 transgenic *Arabidopsis* plants; Figure S2: Subcellular localization of *Bv*REM12 protein in sugar beet protoplasts.
