# Supplementary material for: REM Gene Family in Sugar Beet and Functional BvREM12 in Salt Stress Tolerance
Source: Plants (Basel). 2026 Sep 8;15(18):2745. doi: 10.3390/plants15182745 (PMC13610932; doi:10.3390/plants15182745)
Supplement: Supplementary file 1 [file plants-15-02745-s001.zip › Supplementary Materials Figure S1.pdf]

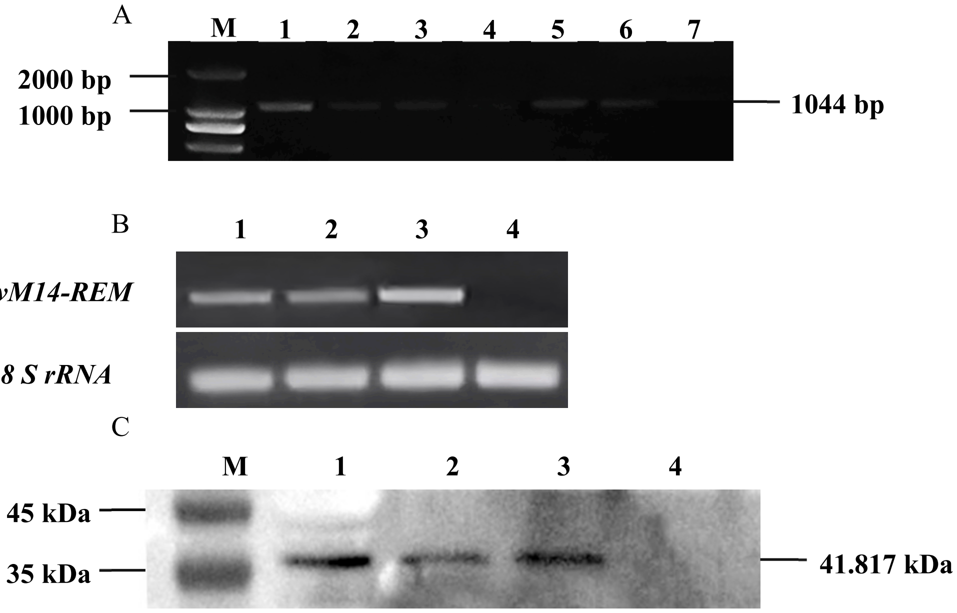

Figure S1. Molecular identification of *BvREM12* overexpressing T3 transgenic *Arabidopsis* plants.(A) PCR amplification of the *BvREM12* fragment from genomic DNA of transgenic lines. M: DL2000 DNA marker; Lanes 1-7: Different independent overexpression lines. The target band was 1044 bp. Subsequently, lines from channels 1, 5, and 6 were sequentially coded as OE1, OE7, and OE10 for subsequent validation. Note: After sampling according to sequential coding, DNA extracted from some plants was contaminated. DNA validation was therefore not performed to avoid false positive results. (B) RT-PCR analysis of *BvREM12* transcription in transgenic *Arabidopsis* lines. Lanes 1-3: Overexpression lines; Lane 4: *Col-0* plant. 18 S rRNA was used as an internal reference gene.(C) Western blot detection of *Bv*REM12 protein. M: Protein molecular weight marker; Lanes 1-3: Overexpression lines; Lane 4: *Col-0* plant. The expected molecular weight of *Bv*REM12 protein was 41.817 kDa.
