# Supplementary material for: REM Gene Family in Sugar Beet and Functional BvREM12 in Salt Stress Tolerance
Source: Plants (Basel). 2026 Sep 8;15(18):2745. doi: 10.3390/plants15182745 (PMC13610932; doi:10.3390/plants15182745)
Supplement: Supplementary file 1 [file plants-15-02745-s001.zip › Supplementary Materials Figure S2.pdf]

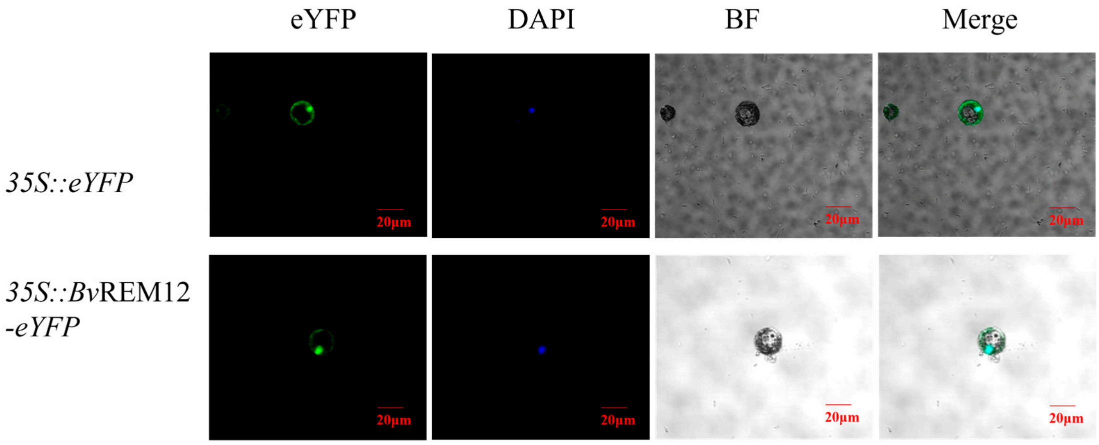

Figure S2. Subcellular localization of *Bv*REM12 protein in sugar beet protoplasts. The subcellular localizations of *Bv*REM12 protein and empty vector in sugar beet protoplasts are shown respectively. The free YFP signal derived from the YFP empty vector control was distributed in the cytoplasm and nucleus. The fluorescence signal of *Bv*REM12-eYFP was detectable in both the plasma membrane and nucleus. The figure includes the eYFP fluorescence channel, DAPI fluorescence channel (nuclear localization dye), bright field channel, and the merged image of bright field, eYFP fluorescence and DAPI fluorescence channels. Scale bar = 20 μm..
