# Supplementary material for: REM Gene Family in Sugar Beet and Functional BvREM12 in Salt Stress Tolerance
Source: Plants (Basel). 2026 Sep 8;15(18):2745. doi: 10.3390/plants15182745 (PMC13610932; doi:10.3390/plants15182745)
Supplement: Supplementary file 1 [file plants-15-02745-s001.zip › Supplementary Materials Table S1.pdf]

Table S1 *BvREM* Gene Family Characteristics

Gene Name	Protein Name	Protein ID	Transcription ID	Gene ID	Chr	Start Position (bp)	End Position (bp)	Length (aa)	Molecular Weight (kDa)	pI	Predicted location(s)
BvREM1	uncharacterized protein LOC104902690	XP_048493696.1	XM_048637739.2	LOC104902690	Chr1	269164	285939	574	63.04	9.77	Cell membrane. Nucleus.
BvREM2	remorin 4.1	XP_010692141.1	XM_010693839.4	LOC104905335	Chr1	53490487	53493356	307	35.32	9.08	Cell membrane. Nucleus.
BvREM3	uncharacterized protein At3g61260 isoform X2	XP_048495011.1	XM_048639054.2	LOC104888676	Chr1	57682649	57686531	174	19.32	8.95	Cell membrane.
BvREM4	remorin 1.4 isoform X1	XP_010672012.1	XM_010673710.4	LOC104888676	Chr1	57682707	57686531	190	21.18	8.92	Cell membrane. Nucleus.
BvREM5	remorin	XP_010667563.2	XM_010669261.4	LOC104884583	Chr3	8864578	8870483	210	23.24	5.99	Cell membrane.
BvREM6	remorin 4.2 isoform X1	XP_010672785.1	XM_010674483.4	LOC104889288	Chr3	49869048	49877236	331	36.13	5.94	Cell membrane.
BvREM7	remorin 4.2 isoform X2	XP_057249131.1	XM_057393148.1	LOC104889288	Chr3	49869503	49877287	329	35.93	5.01	Cell membrane. Nucleus.
BvREM8	remorin	XP_010676488.1	XM_010678186.4	LOC104892299	Chr5	4373931	4376615	196	22.00	7.59	Cell membrane.
BvREM9	remorin 4.1	XP_010678430.2	XM_010680128.4	LOC104893981	Chr5	55562194	55565166	261	30.63	9.59	Cell membrane. Nucleus.
BvREM10	uncharacterized protein LOC104883752 isoform	XP_010666609.2	XM_010668307.4	LOC104883752	Chr6	428209	432903	440	47.68	9.05	Cell membrane. Nucleus.
BvREM11	uncharacterized protein LOC104895366 isoform	XP_010680149.2	XM_010681847.4	LOC104895366	Chr6	7203976	7210570	515	57.28	8.05	Nucleus.
BvREM12	uncharacterized protein LOC104897624	XP_010682851.2	XM_010684549.3	LOC104897624	Chr6	67334287	67337905	347	38.82	8.81	Cell membrane. Nucleus.
BvREM13	uncharacterized protein LOC104899679	XP_010685212.2	XM_010686910.3	LOC104899679	Chr7	51463005	51467622	503	57.02	8.86	Cell membrane. Nucleus.
BvREM14	uncharacterized protein LOC104901250	XP_010687102.1	XM_010688800.3	LOC104901250	Chr8	28325294	28329733	528	59.07	8.86	Cell membrane. Nucleus.
